# Zero-shot deep learning for the annotation of unknown eDNA sequences using co-occurrences and phylogenetic embeddings

**DOI:** 10.1371/journal.pcbi.1013776

**Published:** 2025-12-19

**Authors:** Steven Stalder, Théophile Sanchez, Michele Volpi, Stéphanie Manel, David Mouillot, Arnaud Auber, Morgane Bruno, Virginie Marques, Camille Albouy, Loïc Pellissier

**Affiliations:** 1 Swiss Data Science Center, ETH Zürich and EPFL, Zürich, Switzerland; 2 Ecosystems and Landscape Evolution, Swiss Federal Institute for Forest, Snow and Landscape Research (WSL), Birmensdorf, Switzerland; 3 Ecosystems and Landscape Evolution, Department of Environmental Systems Science, ETH Zürich, Zürich, Switzerland; 4 CEFE, University of Montpellier, CNRS, EPHE-PSL University, IRD, Montpellier, France; 5 Institut Universitaire de France, Paris, France; 6 MARBEC, University of Montpellier, CNRS, Ifremer, IRD, Montpellier, France; 7 Ifremer, HMMN, Laboratoire Ressources Halieutiques, Boulogne-sur-Mer, France; Chinese Academy of Science, CHINA

## Abstract

The advent of environmental DNA (eDNA) metabarcoding marks a transformative era in large-scale biodiversity monitoring. However, the analysis of eDNA datasets is limited by incomplete reference databases and the increasing volume of data requiring processing from raw sequences to annotated taxonomic lists. To curate taxonomic lists from eDNA analysis, geographic constraints are used by expert in post-analysis, which may introduce potential biases in assignments. Instead of relying on expert intervention, a combination of taxonomic and geographic co-occurrences could be directly integrated into machine learning to automatize and improve taxonomic annotation. Here, we introduce a deep learning approach applied to the taxonomic assignment of eDNA sequences, which leverages a species reference database, species co-occurrence data, and a phylogeny to enhance annotation directly from raw sequences. The phylogeny provides the structure to the network’s embedding space in which DNA sequences are placed utilizing an artificial neural network (ANN). We train an additional ANN from the phylogenetic embedding and co-occurrence species data to learn coherent species combinations from the whole collection of eDNA sequences, as opposed to single sequences only. When applied directly to the raw sequences, this method correctly predicts unseen species (i.e., those not contained in the reference database), out of more than 31,000 possibilities, in about 24% of the tested cases by relying on phylogenetic embeddings and geographic modulation. The trained ANNs discern species relationships accurately from the raw data, which facilitates the process of associating sequences with taxa—even those absent from reference databases. When we use real eDNA samples, our predictions mostly agree with those from a traditional bioinformatic pipeline, highlighting the potential of our method for the annotation of the increasing number of eDNA sequences.

## Author summary

The analysis of environmental DNA (eDNA) samples is hindered by the difficulties in assigning DNA sequences to species, which arise from incomplete reference databases. We use deep learning techniques to perform taxonomic assignments even in the absence of known reference sequences for a species. Our method integrates multiple data sources to approach this problem from different angles. We first utilize the information from a phylogenetic tree of fish species to construct a biologically informed embedding space for these species via an optimization problem. We then train an artificial neural network to map known DNA sequences to the correct species in this space. Finally, we use species co-occurrence data to refine our predictions to better correspond to ecological associations as observed in nature. We show that our method is frequently able to assign the correct species from its DNA, even when it has not been recorded in database before. We further validate our method with real eDNA samples, and we show that taxonomic assignments correspond to those made by a traditional bioinformatic pipeline.

## 1 Introduction

DNA, the molecule of life, holds the information necessary for the development and metabolic functioning of organisms, which shape their interactions within ecosystems [1]. Because it is unique to every species, DNA also contains the signal of species taxonomic identity. As organisms interact with their environment, they shed cells and tissues, leaving behind traces of DNA that can be recovered, providing insights into the species composition and functioning of ecosystems [2,3]. By analyzing these DNA traces, referred to as environmental DNA (eDNA), we can identify which organisms are present and what they do within ecosystems [4,5]. Until recently, eDNA analyses were constrained by the high cost of sample sequencing. However, advances in capacity and reduced expenses have shifted the primary challenge to the processing of the ever-growing flux of data [6]. Samples are routinely collected in different ecosystems from multiple sources, such as water, soil, or animal gut contents, generating vast amounts of sequencing data [7]. To transform these raw DNA sequences into actionable information for diagnosing ecological issues, improving animal and human health, or restoring ecosystems, sophisticated data analysis is required [8]. These sequences should be transformed into taxonomic lists that can be associated with ecological or functional properties [3]. One of the current challenges is that the speed at which we can generate DNA data outpaces the development of the reference databases needed to interpret them. Advancements in machine learning offer promising solutions by accelerating the annotation and interpretation of this massive quantity of information by leveraging complementary datasets that can support species annotation [9,10].

The accurate assignment of DNA sequences to a taxonomic identity or functional category is crucial for diagnosing the health of both animals and ecosystems [11], as species composition reflects the degree of environmental degradation [12]. Assessing ecosystem health from DNA data, therefore, requires the accurate taxonomic annotation of raw sequences, enabling the linkage to functional traits stored in databases and facilitating ecological interpretation. To perform taxonomic assignment, eDNA metabarcoding data are typically processed with bioinformatic pipelines that clean raw sequences, remove errors, and match the sequences to reference databases for taxon annotation (e.g., [13]). To achieve this, comprehensive genetic databases are being developed that link DNA sequences to taxonomic names [14] or molecular functions (e.g., metabolic pathways [15] or phenotypic traits). However, reference databases are often incomplete [16], as filling all taxonomic gaps requires considerable time, expertise, and resources for species collection, identification, and sequencing [17]. A new generation of bioinformatic approaches that can incorporate indirect data sources and analytical methods, and improve annotation accuracy, could help overcome the bottleneck created by incomplete reference databases and processing limitations. This, in turn, would ensure that DNA-based diagnostics become more reliable for monitoring ecosystem health and biodiversity [18]. Data associated with DNA sequences exist in various forms, and machine learning (deep learning) offers the potential to exploit these different types of data to derive more accurate species assignments and thus more reliable information on ecosystem health.

Deep learning and artificial neural networks (ANNs) have emerged as flexible tools for integrating multimodal data. These can improve the processing of eDNA sequences in the context of trend analyses, sequence annotation, or evaluations of ecosystem health [19]. Deep learning can be used to ordinate eDNA samples directly from raw data [20], thus facilitating their comparison and highlighting the underlying environmental conditions [21]. It can further be used to learn and predict the association between a sequence and the health of ecosystems [19]. Machine learning relying on K-mers, which are substrings of DNA of length K, has been used to improve the taxonomic assignment process [10] and compare the performance of eDNA markers for species detection [22]. The development of new deep learning architectures, particularly transformers, which rely on layers of attention mechanisms, has revolutionized the field of large language models and made it possible to learn the local association and contextualization of objects [23]. DNA sequences carry phylogenetic signals and conservatism of genes, which can be exploited to refine data to infer taxonomic identity [24]. ANNs can learn to embed DNA sequences according to specific loss functions and learning rules, allowing the placement of sequences within the branches of a phylogenetic tree [25]. This placement would facilitate the representation of relationships between sequences [26] and the final taxonomic assignment. Moreover, ANNs have been used to improve the prediction of species taxonomy from images, integrating information on co-occurring species [27]. In this context, species co-occurrences derived from traditional community surveys could be leveraged by an ANN model to learn the associations between taxa. Generalization across datasets can be enhanced by fine-tuning these models on taxonomically or ecologically similar datasets, a task known as transfer learning. By integrating indirect or ancillary data sources and recent deep learning techniques, in addition to those mentioned above, we can overcome the current limitations of reference databases and make considerable progress in improving the efficiency of eDNA processing for describing species assemblages.

The biodiversity of marine ecosystems is particularly difficult to monitor, due to logistical constraints and the vast expanse they occupy. eDNA metabarcoding has revolutionized the detection of marine biodiversity by reducing the need for traditional, labor-intensive methods such as trawling (e.g., [28]) and underwater visual surveys [29,30]. Compared with conventional techniques, eDNA metabarcoding is particularly effective for monitoring elusive, threatened, or rare species (e.g., [28]). eDNA enables rapid assessment of marine community compositions and offers timely insight into the impacts of anthropogenic stressors, such as pollution, overfishing, and habitat degradation [5,31]. As a result, eDNA is increasingly being integrated into marine biodiversity assessments, providing, for example, a more comprehensive understanding of fish communities and their ecological roles. Beyond species inventories, eDNA-based monitoring generates critical data for conservation efforts by identifying species at risk, evaluating the impacts of human activities, and tracking biodiversity shifts driven by climate change. However, only a fraction of species have corresponding reference sequences in current reference databases for the most common markers used [32], making the identification of sequences to the species level challenging. By facilitating more frequent and large-scale biodiversity assessments, eDNA metabarcoding holds promise for ensuring the sustainable management of marine resources if accompanied by efficient data processing pipelines.

Here, we propose a method that involves combining multimodal data to improve the taxonomic assignment of eDNA metabarcoding sequences. We develop a deep learning algorithm utilizing independent datasets of phylogenies and species co-occurrences to predict taxonomic identifications from sequences, including for species absent from the original reference database. Designed to be fast and simple, our approach processes raw eDNA sequences directly and performs taxonomic assignments after a simple step of sequence clustering. We test the method’s ability to recover the correct taxonomic assignments in this setting and validate it on a global marine eDNA dataset. Furthermore, we compare our method with a traditional bioinformatic pipeline. We ask the following questions:

1. Using phylogenetic embeddings and a neural network trained on DNA sequences, are we able to correctly assign sequences of species that are absent from the reference database?
2. Does using a model trained on co-occurrence information improve taxonomic assignments compared with a baseline model?
3. How well does the output of our method agree with that from a traditional bioinformatic pipeline when applied to a global marine fish eDNA metabarcoding dataset?

## 2 Methods

The multimodal method we present is applied here to a case study on marine ray-finned fish, but it is generalizable to any taxonomic group for which the required data are available. The approach begins with model calibration, which requires several datasets: (i) a reference database of sequences for a specific marker linked to taxonomic names, (ii) a complete phylogeny describing the evolutionary relationships among all species in the focal clade, and (iii) a database of species co-occurrence within local assemblages. In this study, we tested two types of co-occurrence datasets: those inferred from range maps derived from species observations, and those based on direct assemblage observations from diving transects and trawling surveys. The second step involves validating the method using a large global database of eDNA samples collected across multiple marine coastal areas processed with a traditional bioinformatic method.

### 2.1 Data and standard processing

#### 2.1.1 Sequence reference database.

For its training, the model requires information on the association between the genetic DNA sequence and the taxonomic name for at least some of the species. We generate a genetic reference database for the teleo region of the 12s mitochondrial marker by combining sequences downloaded from NCBI and our own sequencing efforts from captured fish individuals. By combining these two main sources of information, we assemble a mitochondrial 12S reference database for the teleo primers to assign a taxonomy to sequences. The reference database covers 9909 sequences from 7445 fish species. During each sampling mission, tissue samples were collected from various fish species, either from fish markets or directly in the field. DNA was extracted and amplified using primers targeting the 12S gene mitochondrial region (V05F-898 5’-AAACTCGTGCCAGCCACC and Teleo-R 5’-CTTCCGGTACACTTACCATG; [33,34]) and sequenced using Sanger sequencing. The 12S reference database contains both the collected species sequences and those from NCBI.

#### 2.1.2 Species phylogeny.

In addition to the reference database, the model incorporates information on phylogenetic relationships among species. Phylogenies represent the evolutionary relatedness of species and are generally reconstructed from genetic sequence data, which may originate from any region of the genome. In this study, we rely on the comprehensive phylogenetic tree of ray-finned fishes compiled by [46]. This backbone tree was first built from genetic sequences and complemented with taxonomic information for species lacking sequence data, thereby maximizing coverage. As a result, this phylogeny represents the most complete and up-to-date information on fish evolutionary relationships. We use this tree to construct a phylogenetic embedding, which allows us to integrate the eDNA reference sequences within a consistent evolutionary framework and establish an explicit link between raw sequence data and taxonomy.

#### 2.1.3 Species co-occurrences.

The other source of information incorporated into the model is the degree of spatial association between species, or co-occurrence, which reflects how frequently pairs of species are found together. In this study, we tested two types of co-occurrence data. The first is derived from range maps, which cover a larger number of species but provide only coarse estimates of association, since they are inferred from overlapping geographic distributions rather than direct observations. The second is based on empirical observations of species co-occurring within the same assemblages. These data provide more accurate information on species associations but are geographically restricted and cover fewer regions and species overall.

First, to generate range-based co-occurrence information, we obtained fish species records from the Ocean Biogeographic Information System (OBIS; http://www.iobis.org). We cleaned the dataset by identifying synonyms based on the WoRMS online database [35] and correcting misspellings. Since OBIS data do not accurately represent the tropical fish assemblage, we also included the Gaspar database, which contains 6,316 tropical reef species [36]. In total, we obtained occurrences for 13,195 strictly marine ray-finned teleost species. We reconstructed the range maps for each species by building a convex polygon surrounding the area where each species had been observed [37]. We then refined each species distribution map by removing areas where the maximum depth fell outside the minimum or maximum known depth range of the species. We aggregated fish range maps on a $1^{\circ }$ grid resolution for all fish species in an equal-area pseudocylindrical projection. For each pair of species, we computed their frequency of co-occurrence based on their ranges.

Second, to generate the assemblage-based co-occurrence, we collected global fish assemblage data from scientific bottom trawl surveys (SBTSs) and visual census surveys. SBTSs involve sampling marine demersal communities that inhabit continental shelves and slopes, using trawls. We used the FISHGLOB dataset [38], a standardized and harmonized dataset of SBTSs containing 216,548 hauls across 29 open-access SBTSs conducted from 1963 to 2020 in subtropical to polar marine continental shelves and slopes of North America and Europe. We complemented this dataset with 107,953 SBTSs conducted in the northeast Atlantic and the Mediterranean Sea, extracted from the DATRAS International Council for the Exploration of the Sea (ICES) database [39] and the MEDITS database for Mediterranean surveys collected from 1983 to 2020 [40,41]. In addition, we collected visual census survey data from 3027 transects of 50 m provided by the Reef Life Survey (RLS) [42] and conducted between 2006 and 2017. We checked the taxonomy of species according to the WoRMS online database [35]. From these combined assemblage data, we computed, for each pair of species, the frequency of their co-occurrence within these assemblages.

### 2.2 Machine learning annotation pipeline

#### 2.2.1 Phylogenetic embedding.

The goal of the proposed approach is to correctly classify sequences of species that are not represented in the reference database, i.e., sequences that the model has not seen during training time. Applying a model trained on these data to previously unseen sequences would lead to a model predicting unrelated species with no sensible accuracy. To address this issue, we introduce an additional source of information that the model could learn to predict taxa for unseen sequences as correctly as possible.

**Fig 1:**
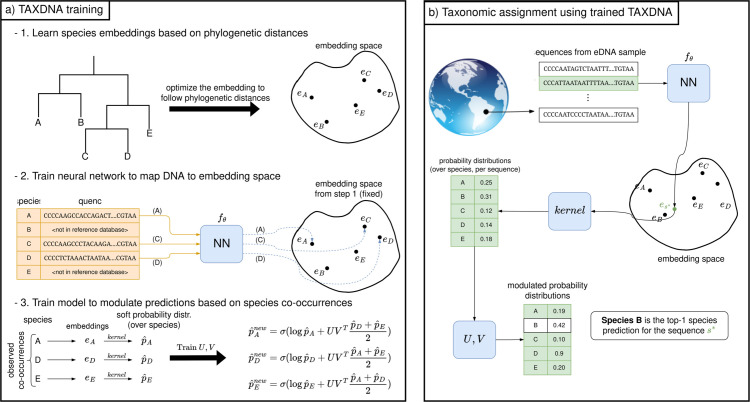
Overview of the machine learning method. (a) During training, the method learns species embeddings from pairwise phylogenetic distances (Sect 2.2.1). We then train an artificial neural network (ANN) to map DNA sequences into the same embedding space, leveraging the previously learned structure (Sect 2.2.2). Finally, we train another ANN model that modulates species predictions based on observed co-occurrence patterns (Sect 2.2.3). (b) Once trained, the model assigns species probabilities to eDNA sequences. Each sequence is first embedded by the trained network, then the kernel converts the position to species probabilities that are modulated by the learned matrices *U* and *V*.

Correctly mapping new, previously unseen DNA sequences to species (i.e., classes) that have not been seen at training time is a task known as zero-shot classification. In our setup, we additionally utilize the species proximity information contained in a phylogenetic tree provided by [46]. We attempt to construct a (latent) embedding space, which is a new data space learned from the phylogenetic tree directly, in which each species in the tree is assigned a vector representation (i.e., the *embedding*). During this learning step, these species embeddings are continuously adapted so that the cosine distance between any pair of embeddings closely matches the normalized phylogenetic distances between the corresponding species in the tree (Fig 1). This can be viewed as a learned approximation of the phylogenetic distance, in this case using the cosine distance, in the form of a numeric vector readily and easily usable in downstream tasks. In a second step, we train an ANN to map DNA sequences into the same embedding space, minimizing the distances between different DNA sequence mappings to their distances as represented by the approximation of the phylogenetic distance (Fig 1).

We consider a data subset of 31,516 species of ray-finned fish. Instead of explicitly learning and optimizing for each pair of species in the tree, we opt for a computationally lighter approach of sampling specific pairs of species. We only learn the embedding for species *i* based on a sampled (methods detailed below) set of other species ${𝒮}_{i}$. The loss function used at every training step to optimize the embedding ${𝐞}_{i}$ from ${𝒮}_{i}$ is as follows:

$${ℒ}_{tree}(i,{𝒮}_{i})=\sum_{j\in {𝒮}_{i}}(d_{tree}(i,j)-d_{cos}({𝐞}_{i},{𝐞}_{j}){)}^{2}$$ (1)

where *e*_*i*_ and *e*_*j*_ denote the learnable embeddings for species *i* and *j* (i.e., what is being learned through the optimization step). $d_{tree}(\cdot ,\cdot )$ denotes the phylogenetic distance between a pair of species defined on a tree, and $d_{cos}(\cdot ,\cdot )$ refers to the cosine distance, which is defined as 1 minus the cosine similarity $s_{cos}(\cdot ,\cdot )$. For two vectors *e*_*i*_ and *e*_*j*_, we have:

$$d_{\text{cos}}({𝐞}_{i},{𝐞}_{j})=1-s_{\text{cos}}({𝐞}_{i},{𝐞}_{j})=1-\frac{{𝐞}_{i}^{T}{𝐞}_{j}}{\|{𝐞}_{i}{\|}_{2}\|{𝐞}_{j}{\|}_{2}}$$ (2)

The embeddings 𝐞 minimizing Eq 1 then receive a label corresponding to each species. Note that these embeddings are optimized directly, i.e., no ANN is used in this part of our method.

Here, we do not loop over all the species and perform pairwise optimization; instead, we choose the set of other species ${𝒮}_{i}$, which determines the optimization of ${𝐞}_{i}$. While we do not want ${𝒮}_{i}$ to be the full set of all species excluding *i*, we want it to be a sample that is representative of the species in the tree with respect to their distance to species *i*. At every branching point in the phylogenetic tree down to the leaf to which species *i* belongs, we sample one species at random from the other branch (i.e., not the one that species *i* is on) and add it to ${𝒮}_{i}$. Therefore, ${𝒮}_{i}$ contains some species that are distantly related to species *i* but also others that are evolutionarily close to species *i*. Although there might be more refined methods based on an explicit search over the distance between nodes in the tree, this approximation works well in practice. Our method is based on stochastic gradient descent, which means that this ‘controlled’ randomization also has the effect that several groups of species with different levels of relatedness can be visited efficiently during optimization.

During the model selection step, we tested several values for the dimensionality of the embedding $𝐞\in {\mathbb{R}}^{d}$. We found that *d* = 64 was the lowest dimensional representation for which we can obtain a small enough (empirically) loss value at convergence, i.e., the cosine distance between embeddings matches the phylogenetic tree distance very closely. Furthermore, this relatively low-dimensional representation guarantees some degree of regularization when optimizing embeddings and prevents overfitting in the subsequent steps. However, an even lower-dimensional space would provide pairwise cosine distances that do not accurately represent the phylogenetic tree, making it difficult to optimize DNA representations that generalize well. We conduct a full hyperparameter search over embedding dimensions, learning rates, and batch sizes (S1 Fig). Because this is an optimization problem on a fixed set of values without an external ground truth or held-out dataset to test generalization, we report the final loss values after 100 training epochs for each experiment, as this directly corresponds to how well the phylogenetic distances are matched. The loss curve clearly flattens after *d* = 64, making it a good tradeoff choice for the reasons stated above. For our final embeddings, we trained over 100 training epochs, where an epoch refers to a full (randomized) pass over all 31,516 species in our phylogenetic tree. We chose a constant learning rate of 0.01 for the AdamW optimizer [47] to optimize the embeddings. With a batch size of 64 and an embedding dimension of 64, a single training run took close to 30 minutes on an NVIDIA P100 GPU.

**Fig 2:**
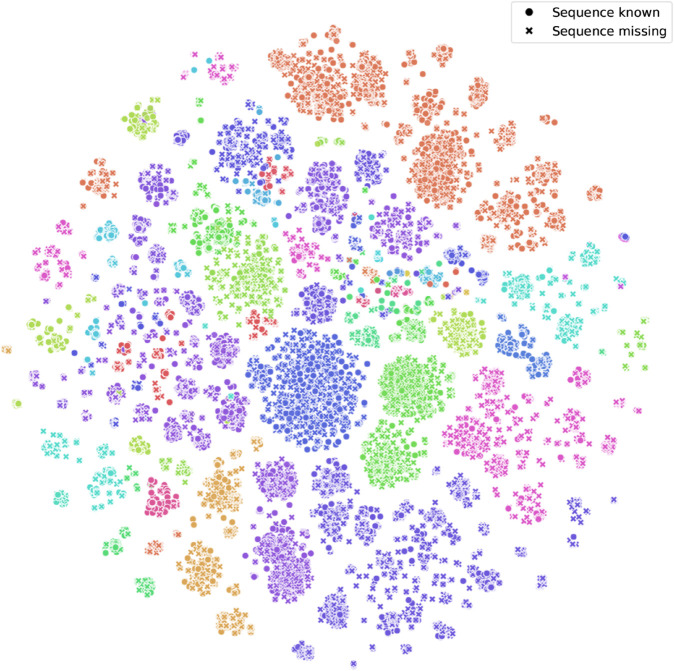
Visualization of the learned embedding space after dimensionality reduction with the t-SNE algorithm. Each point corresponds to a species in the tree, and its color represents its taxonomic order. Points are further distinguished with two different symbols based on whether the species is in the DNA reference database (dot) or not (cross). Axes are omitted because they do not carry an intrinsic meaning.

We used the t-SNE algorithm [48] to reduce the dimensionality for visualization (Fig 2). The color of each point corresponds to the order (referring to the taxonomic rank) of the species represented by the point. We further distinguish the points with two different symbols, which represent whether the corresponding species is in the DNA reference database or not (note that this information was not used for learning the embeddings). While noting that t-SNE dimensionality reduction is only a qualitative representation for high-dimensional data and as such should not be over-interpreted, we observe that the points with the same color tend to be grouped, meaning that we are able to learn embeddings carrying relevant structures based on the phylogenetic information alone. This explains how this learned structure can be leveraged by the other components of the method.

#### 2.2.2 DNA classification.

Once the species embeddings are learned, they are kept fixed and used as an approximation of the phylogenetic tree. This now defines a new representation for species, which we apply as a reference to train an ANN to map raw DNA sequences. Hence, we train a model that takes raw DNA as input and learns a 64-dimensional embedding that approximates the phylogenetic tree as closely as possible, as a representation that captures information similar to that contained the tree. Formally, for a sequence belonging to species *i*, we aim to learn a mapping to the corresponding embedding ${𝐞}_{i}$. We parameterize this mapping by using an ANN that we train on the sequences from a reference database. We train this neural network using a loss function that computes the cosine distance between the learned and fixed phylogenetic embedding ${𝐞}_{i}$ and the mapping of each DNA sequence ${𝐬}_{i}$, for species *i*, by minimizing:

$${ℒ}_{DNA}({𝐬}_{i},{𝐞}_{i};\theta )=d_{cos}(f_{\theta }({𝐬}_{i}),{𝐞}_{i})$$ (3)

where $f_{\theta }$ denotes the neural network with learnable parameters θ. The neural network itself is relatively small. Its first layer is a learnable encoder for the different nucleotide base characters A, C, T, and G (there can sometimes be other characters to denote read errors or ambiguity between the bases). Then, the network applies two one-dimensional convolutional layers, each followed by a rectified linear unit (ReLU) activation. The first convolutional layer uses a kernel size of 5, standard stride and dilation (both equal to 1), and symmetric zero padding of length 2, allowing it to scan across every contiguous 5-mer in the sequence without reducing sequence length. The second convolutional layer also uses a kernel size of 5, but with a dilation rate of 5. In this case, symmetric zero-padding of length 10 is added beforehand so that the output sequence length is preserved. This dilation setting effectively spaces out the receptive field so that the layer does not simply re-learn the local 5-mer patterns detected by the first convolution. Instead, it captures complementary, non-overlapping features at the base-pair level, enabling the model to integrate information across positions that are farther apart in the sequence. Finally, the neural network contains a small multi-layer perceptron (MLP) network head (also called fully-connected layers) to aggregate the information from the convolutional layers into a final embedding for the input sequence. This MLP contains two linear layers separated by another ReLU activation function. It uses a fixed hidden dimension of 2048 and an output dimension that matches the dimensionality of the embeddings, which in our experiments was fixed to *d* = 64. While we experimented with more complex transformer-based architectures and deeper architectures, this simple network worked best with the relatively short sequences we are mapping.

We trained $f_{\theta }$ for a fixed number of epochs while holding out a fixed random set of marine species from the training for evaluating the zero-shot performance of the model (Sects 2.3 and 3.2). A separate validation set for tuning and early stopping was not used because we believed that withholding more data from training was more harmful to performance than not having a validation set for tuning and early stopping. We used a learning rate of 10^−3^ with the AdamW optimizer [47] and a batch size of 16. On an NVIDIA P100 GPU, training one model took between 10 and 20 minutes on average.

#### 2.2.3 Integrating the co-occurrence structure.

The learned embedding space now represents DNA sequences using numerical vectors, which also match the distance between such species on the phylogenetic tree. This has the advantage of transforming a tree and a sequence of characters, complex data representations themselves and hard to work on jointly, into a simple 64-dimensional representation. With this, we can easily perform operations developed for numerical data, while still jointly working on a phylogenetic tree approximation and DNA data. For example, operations such as retrieval by nearest neighbor can be performed by calculating which species embedding ${𝐞}_{j}$ is closest to ${𝐞}_{i}$, and using the corresponding species *j* as the output of our model as the most likely label for species *i*. We could also easily perform k-nearest neighbors and majority voting, to estimate frequencies and the most common match. In this work, we transform our sequence embedding into probability vector estimates over species by using a kernel function relying again on the cosine distances, de facto performing soft nearest neighbor aggregation. The predicted probability of a species *i*, given an unlabeled sequence ${𝐬}^{*}$, is modeled as follows:

$$P(i|{𝐬}^{*})=\frac{k(f_{\theta }({𝐬}^{*}),{𝐞}_{i};\tau )}{\sum_{j\in 𝒮}k(f_{\theta }({𝐬}^{*}),{𝐞}_{j};\tau )}\;\text{with}\;k({𝐱}_{1},{𝐱}_{2};\tau )=\exp(\frac{-d_{cos}({𝐱}_{1},{𝐱}_{2})}{\tau })$$ (4)

where τ is a temperature parameter, which we set to 0.05, and 𝒮 is the set of all species in our tree. We modulate all of the prediction jointly, such that the combined prediction of species in a community matches our prior knowledge from an ecological standpoint. We use species co-occurrences to train an additional model that transforms our initial probability distribution vectors into improved ones that encode species co-occurrences and hence are more likely to match the observations.

From the various datasets on species co-occurrences included in our study, we know that, at a given site, a set of *M* species $\left\{i_{1},\ldots ,i_{M}\right\}$ co-occur. Let $\left\{{𝐞}_{1},\ldots ,{𝐞}_{M}\right\}$ be their learned embeddings. Although we know the co-occurring species at each site exactly, we transform these embeddings to probability vectors $\left\{{\hat{p}}_{1},\ldots ,\hat{p_{M}}\right\}$, as we did for the case described above for DNA sequences in Eq 4. We then transform these initial estimates of probability vectors into ones encoding species co-occurrences, denoted as ${\hat{p}}^{co}$, via a simple linear transformation:

$${\hat{p}}_{i}^{co}=\sigma (\log{\hat{p}}_{i}+UV^{⊤}\frac{1}{M-1}\sum_{l=1,l\neq i}^{M}{\hat{p}}_{l})$$ (5)

where σ is the softmax function and $U,V\in {\mathbb{R}}^{d\times m}$ are matrices of learnable parameters, where *d* is the total number of species in our full tree and *m* is a freely adjustable parameter that we set to 256 for our experiments. *U* and *V* can be optimized by minimizing the negative log-likelihood of the correct species in the modulated probability vectors:

$${ℒ}_{co}(k)=-\frac{1}{M_{k}}\sum_{i=1}^{M_{k}}\log{\hat{p}}_{i,y_{i}}^{co,k}$$ (6)

where *y*_*i*_ denotes the index of species *i* in the probability vector and *k* now specifies a given location. We iterate through all of these locations over the course of many training steps. This model learns relevant co-occurrence patterns for various pairs of species by spanning all sampled locations. We model co-occurrences in this way because DNA sequences are not available for all species, whereas phylogenetic embeddings are available for all of them. While we use these species embeddings during training, this model can, at inference time, be directly used with co-occurring DNA sequences by simply parsing DNA sequences to obtain the probability of occurring species.

To train this model, we use a constant learning rate of 0.001 and the AdamW optimizer [47]. The training batches are of varying size, depending on the number of co-occurring species at a site. We do not use further batching over multiple sites for each training step. Our training runs spanned from 30 minutes to 2.5 hours on an NVIDIA P100 GPU, depending on whether we trained the model on co-occurrence patterns from observed communities or those only inferred from range maps, which resulted in different amounts of training data. Because this is an optimization problem over observed co-occurrences without any notion of generalization to unobserved co-occurrences, there are no validation or test splits here.

### 2.3 Internal and external validation

To simulate how well our method would generalize to DNA of species not in the reference database, we hold out a random set of 431 marine fish species during the training of our DNA model. We then evaluate the method’s classification accuracy on that set at test time (see Sect 2.2.2 for more information on the training setup). We calculate the percentage of correctly predicted species over all predictions made, where the ‘predicted’ species is simply the one with the highest probability (*i.e.,* the one closest in the embedding space). We also evaluate the percentage of predictions that correspond to the correct genus, family, and order. The results are first averaged *per species* (over all known sequences of each species, which we independently process), after which we average these per-species results over all species. We randomly select 431 species from the intersection of species in our phylogenetic tree and those observed in true communities and contained in the range maps. This allows a comparison between the method with and without the co-occurrence model, as well as between the two co-occurrence datasets used.

To evaluate the contribution of our co-occurrence model, we test our method to classify both single sequences, with and without the co-occurrence model. For the data on co-occurring species, we either add their sequence mappings as provided by our DNA model, if at least one DNA sequence was known, or transform their embeddings available for all species with noise. In the second case, we add noise to the embeddings because using the embeddings directly would correspond to the unrealistic case in which we can perfectly map DNA sequences to the correct species embedding. We add multivariate white Gaussian noise to these embeddings, for which we tune the variance to be in the range of the errors that our model makes with sequences from species it has not seen at training time. As an alternative to this setup, we also experimented with completely discarding the information on co-occurring species without known sequences, which led to only minor differences in the results.

We perform this evaluation using two different co-occurrence datasets: empirically derived co-occurrence data, representing actual observations where species were recorded together at the same location (which we call the *community dataset*, see Sect 2.1.3), and co-occurrences inferred from range maps (which we call the *range maps dataset*, see Sect 2.1.3). For the observation-based co-occurrences, we directly train our model on the data from each recorded site and the species observed there. For the co-occurrences inferred from range maps, we treat any two species as co-occurring if their range maps overlap at the same latitude-longitude grid cell of $1^{\circ }$ resolution. In addition, we train the co-occurrence models in the same way as described in Sect 2.2.3. We test the two co-occurrence models, each trained on one of the two datasets, as well as a model trained on the data from both datasets.

While the main contribution of our method is the ability to classify sequences of new species that have not been seen by the model at training time, we also compute results for the DNA of species that the model saw during training, as further validation. Machine learning models are generally expected to fit the training set well, often arbitrarily close to perfect, while assessing the model on data not seen during training (held out) is key to assessing the model generalization. Therefore, metrics computed on the training set are usually omitted. In our case, one can expect to encounter several sequences of species at test time that are also contained in the training set, so these results do have some relevance. We therefore repeated the same experiment outlined above, but with all the species contained in the training set (results presented in Sect 3.1). The main results on new species (held out during training) are presented in Sect 3.2.

#### 2.3.1 Environmental DNA sampling and analyses.

The main purpose of the model is to annotate sequences from eDNA metabarcoding data. For this purpose, a global dataset of 369 eDNA samples collected at 100 stations from 26 sites (groups of stations were separated by at least 35 km) was assembled. Two different sampling methods were used: a 2-km sampling transect of 30 L (surface or bottom depth) or point samples of 2 L (see [43]). Filtration was done with polyethersulfone filters (0.2 μm pore size), and samples were processed following the protocol proposed by [22], in a dedicated DNA laboratory equipped with positive air pressure, UV treatment, and frequent air renewal. Amplifications of metabarcode sequences were done using a teleost-specific 12S mitochondrial rRNA primer pair (teleo, forward primer ACACCGCCCGTCACTCT, reverse primer CTTCCGGTACACTTACCATG; [34]) that amplifies a region of 64 base pairs on average (range 29–96 bp) of the mitochondrial 12S region. The teleo marker is appropriate for fish studies, owing to its high interspecific variability and short length [22]. We performed 12 individually tagged DNA amplifications (via polymerase chain reaction, PCR) per sample. Various sequencers and methods were used for the sequencing (details given in [44]).

#### 2.3.2 Annotation with standard pipeline.

We performed the bioinformatic processing of raw eDNA sequences with paired-end merging using VSEARCH, sequence clustering using SWARM [45] (with default parameters), and chimera removal using UCHIME. Taxonomic assignment of molecular operational taxonomic units (MOTUs) was done using the lower common ancestor (LCA) algorithm ECOTAG, implemented in the OBITOOLS toolkit [13]. Methodological details are described in [44]. Taxonomic assignments obtained from the LCA algorithm at the species level were accepted if the percentage of similarity with the reference sequence was 100%, at the genus level if the similarity was between 90% and 99%, and at the family level if the similarity was between 85% and 90%, following previous studies, to avoid over-confident assignments. If these criteria were not met, the MOTU was discarded. The data was further curated by removing all occurrences with fewer than 10 sequences per sample and MOTUs occurring only once.

### 2.4 Application to eDNA data

As further validation, we compared the predictions of the proposed machine learning method to a standard bioinformatic pipeline (see Sect 2.3.2), by computing taxonomic assignments on the same eDNA samples. Before applying the ANN annotation, we first pre-processed the raw sequences of each sample by removing primers and tags and running the SWARM [45] algorithm, using the default parameters and keeping clusters of size ≥3. SWARM allows clustering of sequences into OTUs using a local, iterative, single-linkage process to group closely related sequences. While SWARM processing might itself introduce certain biases, we applied it to remove redundancy and non-informative noise, to reduce the computational load when using our method. It is possible to just run the machine learning pipeline on every demultiplexed raw sequence without SWARM processing, but this requires running hundreds of thousands of mostly redundant sequences per sample through our model, which is slow and hinders our method’s easy applicability with standard resources. After SWARM processing, we ran all remaining sequences through our trained machine learning models at the same time, resulting in a predicted species for each. Running this pipeline, including SWARM processing and our machine learning method, took only a few seconds per eDNA sample, even on a standard CPU. We compared our results using eDNA data with those obtained through the traditional pipeline. A few sequences for which predictions were made did not match at all, due to slightly different experimental setups. Hence, we only evaluated the agreement for sequences that were assigned to any taxonomic rank by both our method and the traditional pipeline. We computed results for a total of 369 eDNA samples from 10 different regions.

## 3 Results

### 3.1 Internal validation

We first present the results obtained for DNA sequences of species included in the training set (Table 1). These experiments follow the procedure described in Sect 2.3, but only predictions for species used during training are shown; results for held-out species are reported in Sect 3.2.

**Table 1:** Top-1 accuracy for the predictions of species with DNA sequence seen during training, as well as for their corresponding genus, family, and order.

Evaluation Method	Species (31,516)	Genus (4,819)	Family (515)	Order (67)
Single Sequence	75.38%	90.13%	97.51%	99.70%
Co-Occ. (Community Model)	67.44%	86.60%	96.29%	99.68%
Co-Occ. (Range Maps Model)	66.34%	84.90%	96.38%	99.39%
Co-Occ. (Combined Model)	65.69%	84.56%	96.29%	99.41%

The row labeled ‘Single Sequence’ refers to the results obtained using only the DNA model based on the tree embedding, without incorporating a co-occurrence model. Rows starting with ‘Co-Occ.’ indicate results when the co-occurrence model in brackets was used to modulate the predictions from the DNA model. The ‘Combined Model’ was trained with both co-occurrence data from true communities and co-occurrence data inferred from range maps. The numbers in brackets below the column headers indicate the number of known unique values in our data for that column.

The row labeled ‘Single Sequence’ refers to the results obtained using only the DNA model based on the tree embedding, without incorporating a co-occurrence model. Rows starting with ‘Co-Occ.’ indicate results when the co-occurrence model in brackets was used to modulate the predictions from the DNA model. The ‘Combined Model’ was trained with both co-occurrence data from true communities and co-occurrence data inferred from range maps. The numbers in brackets below the column headers indicate the number of known unique values in our data for that column.

Our method classified known sequences with high accuracy. While the correct species and genus were predicted with up to 75% and 90% accuracy, respectively, their corresponding family and order were almost always correct, with accuracies of 97.5% (family) and 99.7% (order). These numbers are in line with what one would expect using traditional methods because correct species-level assignment is sometimes hindered by shared sequences across different species [22]. Using any co-occurrence model (trained on true communities, on co-occurrences inferred from range maps, or both) slightly reduced the accuracy compared with only using the DNA model. The added complexity of the co-occurrence model may have perturbed some of the already good initial predictions. The difference in accuracy was still relatively minor, though, at least when the best co-occurrence model based on the observation-based co-occurrences was used.

### 3.2 External validation

The zero-shot classification results, i.e., for species not seen by the model during training (Table 2), are based on the experimental setup described in Sect 2.3. The row labeled ‘Single Sequence’ shows the accuracy of our model when DNA sequences were mapped with our DNA model, without using the co-occurrence model and data on co-occurring species. What is striking is that this model rarely predicted the right species (i.e., the mapping of the DNA with the model was not closest to the true species in the embedding space), yet the predicted species often had the same genus, family, or order as the true species. Hence, the DNA model alone already made relatively good predictions, and the initial probabilities only needed to be slightly adjusted by our co-occurrence model to predict correctly at the species level.

**Table 2:** Top-1 accuracy for the predictions of species with DNA sequence not seen during training (zero-shot), as well as for their corresponding genus, family, and order.

Evaluation Method	Species (31,516)	Genus (4,819)	Family (515)	Order (67)
Single Sequence	0.70%	50.70%	80.70%	86.74%
Co-Occ. (Community Model)	24.23%	56.23%	80.50%	89.05%
Co-Occ. (Range Maps Model)	14.44%	55.68%	80.89%	88.15%
Co-Occ. (Combined Model)	13.94%	55.13%	80.95%	88.23%

The row labeled ‘Single Sequence’ refers to the results obtained using only the DNA model based on the tree embedding, without incorporating a co-occurrence model, is used. Rows starting with ‘Co-Occ.’ indicate results when the co-occurrence model in brackets was used to modulate the predictions from the DNA model. The ‘Combined Model’ was trained with both co-occurrence data from true communities and co-occurrence data inferred from range maps. The numbers in brackets below the column headers indicate the number of unique values in our data for that column.

Regardless of the data the co-occurrence model was trained on, i.e., ‘true community’ and/or ‘range maps’, the benefits of using a co-occurrence model are evident. Although the scores for genus, family, and order only improved slightly, the correct species was predicted significantly more frequently. The best model combination predicted the right species—for DNA sequences that had not been seen before—out of more than 31,000 possibilities in more than 24% of cases, and it matched the right genus, family, and order in 56%, 81%, and 89% of cases, respectively. The model relying only on co-occurrence data from the ‘community’ dataset provided the best results overall, outperforming the model based on co-occurrences inferred from range maps.

**Fig 3:**
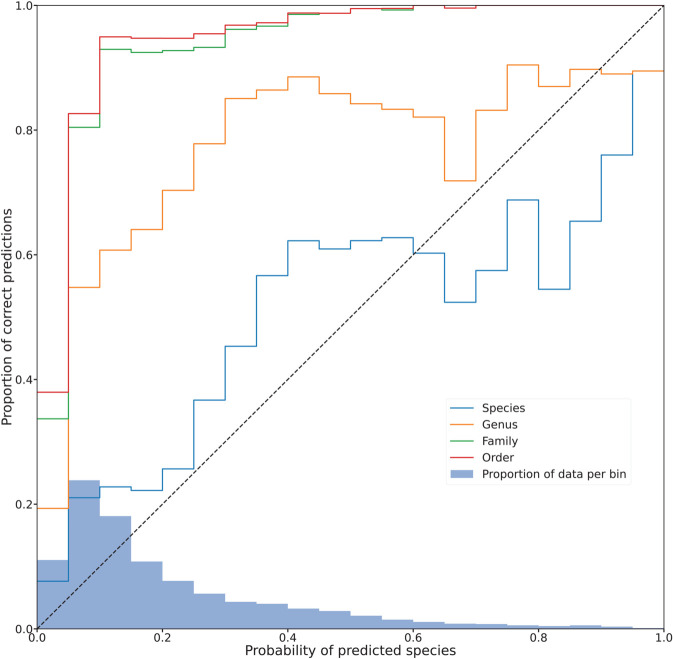
Calibration strength of our model outputs. The predicted species output probability is shown on the x-axis, and the corresponding proportion of correctly predicted assignments at the species, genus, family, and order level is shown on the y-axis. The dashed black line represents a perfect calibration for the solid blue (species) line. The bar plot at the bottom depicts the respective proportion of predictions that fall into each (5%) probability bin.

When making predictions, our method assigns a probability to each species, but these values do not correspond to a real, calibrated probability representing exactly to how often the prediction is correct. We investigated this relationship (Fig 3), and we observed a strong correlation between the predicted species probabilities and the accuracy of the predictions at the species level, indicating that this score reflects the likelihood of occurrence. The probabilities are relatively low for most of the top species. Since many species are very similar, a model should distribute probabilities to a likely set of species, rather than making overconfident predictions for a single one.

### 3.3 Comparison with a traditional pipeline applied to eDNA samples

**Fig 4:**
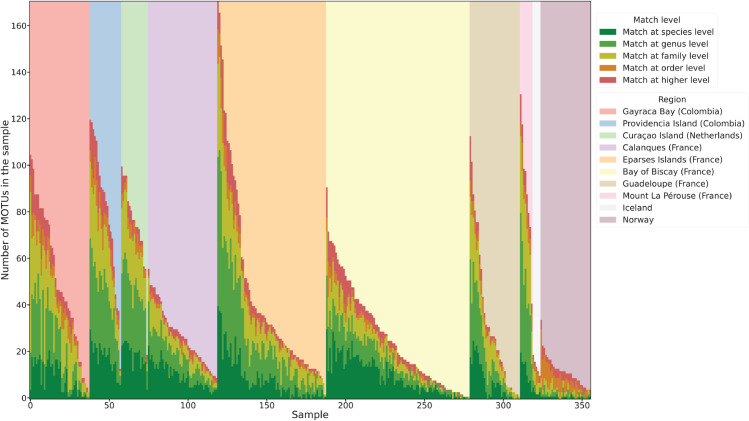
Comparison of predictions for eDNA samples using our method and the traditional pipeline. Predictions are compared at different taxonomic levels and grouped by region. Each vertical line represents the results for one eDNA sample.

Based on the experiments described in Sect 2.4, we compared the number of MOTUs predicted with our method and the predictions from the traditional pipeline applied to the eDNA samples (Fig 4). Across all marine regions, our predictions and those from the traditional pipeline often matched at the species (23.7%), genus (31.3%), family (25.8%), or order (7.6%) level, with mismatches occurring only infrequently (11.6%). These numbers were also well-balanced across the marine regions, with only the samples from Norway resulting in considerably fewer matching predictions at the species level. Moreover, many of the predictions that only matched at higher levels were because the traditional pipeline provided predictions at these higher levels, whereas our method always predicted at the species level. Among all predictions that matched at any level, the traditional pipeline provided a prediction at the species level in 42.4%, at the genus level in 37.2%, at the family level in 17.3%, and at the order level in 3.1% of the cases.

## 4 Discussion

The volume of eDNA metabarcoding data is growing exponentially [49], as more laboratories acquire the capacity to process these samples and the cost of sequencing decreases [50], enhancing our ability to monitor biodiversity. The assignment of species names to sequences is essential for linking them to functional traits, phylogenetic information, and conservation status, as well as for detecting potentially invasive species [3]. Here, we applied machine learning for the taxonomic assignment of eDNA sequences directly on demultiplexed raw data to minimize pre-processing steps and speed up the analysis [20]. Our approach integrates DNA sequence data with phylogenetic embeddings and co-occurrence information through an approach involving ANNs. Our results demonstrate that incorporating and modeling this complementary information enhances predictive accuracy. For species encountered during the training phase, our method achieved performance levels comparable to those of traditional bioinformatic pipelines, while offering improved efficiency and user-friendliness, from the raw sequencing data at the sample level to the final species list. Although the current dataset used to calibrate the ANN is extensive, the ongoing collection of eDNA samples and expansion of the reference database are expected to further improve the accuracy of this approach. Given the challenges in achieving a complete reference database in the near future, machine learning based annotation methods will be indispensable for maximizing the utility of eDNA data in the meantime.

The taxonomic assignment of sequences is often limited by incomplete reference databases [32], which precludes the identification of sequences from unreferenced species. Our results indicate that phylogenetic embedding allows better prediction of taxa when provided with the full species assemblage information. In ecosystems, species assemblages comprise multiple lineages and, due to their diversification histories, are inherently phylogenetically diverse [51]. Speciation with geographic segregation is the predominant mode of species diversification, and closely related lineages are geographically dissociated [52]. Assemblages therefore usually contain a single representative per lineage, rather than clusters of closely related species, resulting in phylogenetic dispersion. We leveraged this characteristic by incorporating phylogenetic embeddings that position sequences within an embedding space representing evolutionary space [53]. Our method can pinpoint the most likely species among similar taxa by modulating the initial DNA-based placement within these embeddings according to co-occurrence data. Our approach correctly labeled previously unseen species (i.e., species absent from the genetic reference database) in approximately 25% of the cases when community information was incorporated, compared with less than 1% when co-occurrence data were not used. The algorithm’s performance may be worse for assemblages characterized by in situ diversification, where many closely related species coexist [54], leading to context-dependency in the method’s performance for species-level assignments. Assignments at higher taxonomic levels, such as genus or family, should not be greatly affected by this issue. Our approach demonstrates how machine learning can enhance species annotation by providing the means to overcome incomplete reference data in eDNA studies [16].

The quality of the complementary information used in our method is critical, as these data substantially influence the performance of the taxonomic assignments. When derived from a range mapping algorithm, co-occurrence data represent potential species co-occurrences, whereas empirically derived co-occurrence data represent observations where species were recorded together at the exact location, which generate informative co-occurrence networks [55]. For our method, co-occurrence information derived from assemblages proved to be more effective than associations inferred from range maps. Generally, range map models are generated from single-occurrence records by learning species–environment associations and projecting distributions on a regular grid [56]. When several species are predicted to be suitable in a given grid cell, they are assumed to co-occur. However, modeled range maps face several limitations, including uncertainties in individual observations, challenges in selecting and resolving environmental variables, and the general omission of biotic interactions [57]. Consequently, co-occurrence data inferred from such models are more prone to inaccuracies compared with direct observation of co-occurrence [58].

More advanced ANN-based approaches that involve modeling the joint probability of species occurrences and better integrate biotic interactions exist; these approaches could help improve the accuracy of inferred co-occurrence in the future [59]. In our work, we incorporated major community datasets from SBTSs and the RLS, which were readily available and led to improvement compared with data inferred from range maps. However, much of the information on species co-occurrences is present in various datasets and publications, but remains underutilized or difficult to access. Consolidating and incorporating these datasets could further enhance the predictive power of our approach. Our study highlights the critical role of data quality, digitization, and curation in feeding computational models, a principle central to many machine learning applications in other domains. Future efforts should involve integrating diverse, high-quality empirical data and refining ANN models to better capture complex ecological interactions, ultimately leading to more accurate and robust species annotation for eDNA datasets.

Our proposed method reliably predicts species composition from eDNA samples, with results comparable to those obtained using traditional bioinformatic pipelines. Unlike conventional approaches, our algorithm performs seamless taxonomic assignment from raw demultiplexed eDNA sequences. Given the large volume of sequence data, we implement a clustering step before analysis, ensuring that the method remains efficient and scalable even for samples with considerable sequencing depth, i.e., those that contain millions of sequences.

The performance of our approach differed between regions, consistent with previous studies using traditional bioinformatic pipelines [60]. For example, predictions in the Arctic were characterized by lower confidence, likely due to the limited reference database and the occurrence of multiple recent species radiations [61], which result in highly similar sequences that complicate species-level assignment. In contrast, the reference database is more comprehensive along the Atlantic coast of France [62], with robust co-occurrence data collected over more than 20 years during scientific campaigns, leading to predictions that were consistent between methods.

We have developed a user-friendly application which demonstrates that, once trained, the pipeline can classify species from an eDNA sample (approximately 200 MB in size) within a few seconds on a standard CPU. Given the modest computational demands of our method, the tool can be easily deployed on cloud platforms, increasing its accessibility to laboratories worldwide for global biodiversity monitoring projects [63]. Here, we presented an accessible and efficient method for annotating raw eDNA data that not only matches the performance of traditional pipelines but also benefits from the integration of independent data to make predictions beyond previous capabilities. As more independent data become available [64], we anticipate that the accuracy and reliability of our method will continue to improve, further advancing biodiversity monitoring efforts.

Although our current results are promising, several limitations remain. First, our approach would benefit from more comprehensive and geographically diverse co-occurrence data, to improve the handling of ‘unknown’ species not represented in existing datasets. Second, the phylogenetic tree used in this study introduces some limitations. We relied on the phylogeny provided by [46], which is constructed from authentic sequence data for only about half of the species; for the remaining species, branch placements are inferred based on taxonomic information. This mixed approach can reduce the resolution of certain clades and, consequently, may have affected the accuracy of our phylogenetic embeddings. Moreover, basing our analysis on a single phylogenetic tree—when 100 were available after species regrafting—led to an inherently uncertain model and thus added another layer of potential error that should be addressed in future work. In addition, our current pipeline integrates several components: a phylogenetic tree, a model trained on DNA sequence data, and a separate model trained on co-occurrence data. While we have made this multi-model pipeline readily accessible for ray-finned fish on the teleo marker, extending it to other taxonomic groups would require training multiple models for each new taxon and new eDNA marker [65]. Future work may involve developing an ensemble of ANNs tailored to each marker and taxonomic group, thereby creating a family of models to choose from when processing eDNA filters.

Despite these challenges, our method offers major advantages. Once trained, the pipeline can rapidly classify species from eDNA samples, making it fast and highly accessible even to individuals with limited bioinformatic skills. Moreover, our approach provides probability scores for each taxonomic assignment, allowing users to assess the confidence of the predictions. This confidence ranking is particularly valuable, as it helps to mitigate potential misinterpretations in taxonomic assignments [60]. Together, these strengths underscore the potential of our approach to advance eDNA analyses, even as we continue to refine and expand its components for broader application.

## 5 Conclusion and outlook

Our method represents a step towards automation of eDNA sequence processing, converting raw sequences into comprehensive taxonomic lists. By integrating phylogenetic and co-occurrence information, our method not only achieves rapid species identification for species with known DNA, but also enables predictions beyond the DNA reference database. Our method further lays the groundwork for coupling machine-learning-based taxonomic assignments with other machine-learning models, including large language models. This integration will enable the swift translation of taxonomic lists into meaningful ecological interpretations, such as assessments of anthropogenic impacts on biodiversity [19]. While our current application is focused on marine ecosystems, the underlying framework is broadly applicable to any taxa with available phylogenetic and community data, such as vertebrates [66], plants [67], and some groups of invertebrates [68]. Together, these innovations open new avenues for biodiversity monitoring and ecological assessment, promising a transformative impact on how we use eDNA data in both research and conservation efforts.

## Supporting information

S1 Fig Hyperparameter analysis for phylogenetic embedding. (PDF)
