# Supplementary material for: Zero-shot deep learning for the annotation of unknown eDNA sequences using co-occurrences and phylogenetic embeddings
Source: PLoS Comput Biol. 2025 Dec 19;21(12):e1013776. doi: 10.1371/journal.pcbi.1013776 (PMC12747428; doi:10.1371/journal.pcbi.1013776)

Zero-shot deep learning for the annotation of unknown eDNA sequences using co-occurrences and phylogenetic embeddings

Steven Stalder[1], Théophile Sanchez[2,3], Michele Volpi[1], Stéphanie Manel[4,5], David Mouillot[6], Arnaud Auber[7], Morgane Bruno[4,5], Virginie Marques[2,3], Camille Albouy[2,3], Loïc Pellissier[2,3*]

1 Supporting Information

S1 Fig Hyperparameter analysis for phylogenetic embedding. Loss vs. embedding dimensionality (D) for different learning rate (LR) and batch size (BS) values.

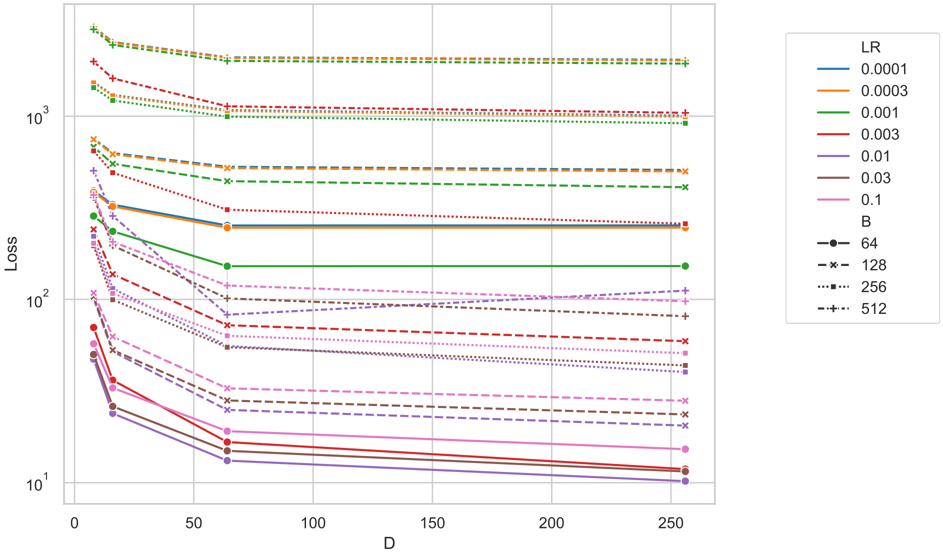

Supplement: S1 Fig — (PDF) [file pcbi.1013776.s001.pdf]
